# A multifactor approach to forecasting Romanian gross domestic product (GDP) in the short run

**DOI:** 10.1371/journal.pone.0181379

**Published:** 2017-07-24

**Authors:** Daniel Armeanu, Jean Vasile Andrei, Leonard Lache, Mirela Panait

**Affiliations:** 1 Department of Finance, The Bucharest University of Economic Studies, Bucharest, Romania; 2 Business Administration Department, Petroleum-Gas University of Ploiesti, Ploiesti, Prahova, Romania; 3 Cybernetics, Economic Informatics, Finance and Accounting Department, Petroleum-Gas University of Ploiesti, Ploiesti, Prahova, Romania; East China University of Science and Technology, CHINA

## Abstract

The purpose of this paper is to investigate the application of a generalized dynamic factor model (GDFM) based on dynamic principal components analysis to forecasting short-term economic growth in Romania. We have used a generalized principal components approach to estimate a dynamic model based on a dataset comprising 86 economic and non-economic variables that are linked to economic output. The model exploits the dynamic correlations between these variables and uses three common components that account for roughly 72% of the information contained in the original space. We show that it is possible to generate reliable forecasts of quarterly real gross domestic product (GDP) using just the common components while also assessing the contribution of the individual variables to the dynamics of real GDP. In order to assess the relative performance of the GDFM to standard models based on principal components analysis, we have also estimated two Stock-Watson (SW) models that were used to perform the same out-of-sample forecasts as the GDFM. The results indicate significantly better performance of the GDFM compared with the competing SW models, which empirically confirms our expectations that the GDFM produces more accurate forecasts when dealing with large datasets.

## Introduction

Modelling the short-term dynamics of real GDP is paramount to economic policy. Of the many statistical approaches that have been used in this area recently, principal components analysis stands out as a preferred choice because it integrates large sets of variables in frameworks that rely on only a few common factors used to produce nowcasts and forecasts of economic output.

Forecasting economic output and understanding the main drivers of output dynamics are paramount to economic policy making. In today’s complex and highly connected economies we are faced with a discouraging amount of data linked to the processes that determine the fluctuations of GDP. While it is not possible to determine a priori whether this huge amount of data should be entirely used in the decision making process, it is also obvious that one cannot afford to ignore the complex macroeconomic relationships that are based on the correlations between output and at least some of these variables.

As highlighted in [1], most empirical analyses of monetary policy are based on the (somewhat inappropriate) assumptions that policy makers use limited amounts of information, despite being clear that policy makers exploit overwhelming numbers of data series. The mere fact that policy makers choose to not ignore variables that are not obviously relevant for the purpose of modelling the processes of interest implies that there is value in the data that may improve significantly the forecasting exercise. The same reasoning applies to the endeavor of forecasting economic growth, in which overreliance on superficial but easily understandable methods (i.e.,vector autoregressions) could turn out to be a risky business due to the very large number of economic and noneconomic variables that influence economic growth.

In this paper we aim to develop a dynamic multifactor model that can be used to forecast the short term dynamic of Romania’s real GDP using a large dataset of predictors. The model is based on the modelling framework developed by [2];[3]. As we shall discuss later, the dynamic multifactor model uses spectral density analysis to assess the dependence of GDP on several economic and noneconomic variables and subsequently produces forecasts (or, if needed, nowcasts) of GDP based on the generalized principal components technique. Afterwards, we propose a alternative model building on the work of [4];[5] and based on the mechanics of standard (static) principal components analysis. This model is similar in concept to the dynamic multifactor model but, as we shall see later, it has some limitations. Nevertheless, we provide it in order to assess empirically whether resorting to complex dynamic data analysis leads to an improvement in performance compared with conceptually simpler models.

This manuscript is structured as follows. In the ensuing section it is provided a brief review of the literature that deals with factor models and their applications in the process of estimating economic variables and then we discuss the concepts underpinning the two models that we have developed. The third section contains a case study in which we focus on the application of our two models to forecasting the dynamics of Romania’s real GDP. An extensive analysis will be provided here, as well as a performance comparison between the competing models. The fourth section concludes and discusses further action to be taken in order to improve the performance and the accuracy of the models.

## Literature review

In the current context of the availability of huge numbers of time series (both soft and hard, aggregated and disaggregated, and with mixed frequencies) from a large number of sources (central banks, statistics offices, supranationals, as well as professional services providers), it is normal to address the problem of summarizing the time series in the most effective way possible for the purpose of analyzing and forecasting the relevant processes. To this end, factor analysis is the most popular choice because it is based on a simple principle [6]. Any variable can be decomposed into the sum of two orthogonal (i.e., uncorrelated) components–one common component that is highly correlated with the data panel and one idiosyncratic (i.e., specific) component. The main idea is that the common component is driven by a relatively small number of latent, unobservable variables (the “common” factors) that are linear combinations of the variables in the initial data set.

Traditional factor analysis (i.e., exact factor models) is based on the restrictive assumption of serial uncorrelation between the idiosyncratic components [7]. One direct consequence of this restriction is that the application of strict factor models to complex economic processes has limited effectiveness, as this approach is unlikely to be realistic in the context of complex real-world economic processes [4]. However, strict factor models form an important part of the relevant literature [8–11]. In the case of small data panels, the model can be estimated using maximum likelihood [6]. However, for large factor models (in which the number of variables exceeds 20; see [7] for further details), experience shows that the convergence of the standard maximization algorithms is slow. The issue can be solved by switching to estimation techniques based on principal components analysis which has recently gained wide acceptance among economists as a reliable forecasting tool [12]. However, principal components analysis is used in a variety of other applications in the field of finance, such as estimating the factors that determine asset returns (see, for example, the discussion in [13] and [14]), computing coincident and leading indicators of economic activity by exploiting both cross-country and within-country correlations in a dynamic framework, as detailed in [15].

Several studies have shown that the fairly restrictive and unrealistic assumption of orthogonality of the idiosyncratic errors can be relaxed if the number of variables in the model is large, i.e. it tends to infinity [5];[16];[17]. These factor models are called approximate factor models and allow for (i) the weak serial correlation of the residuals (i.e., idiosyncratic errors), (ii) the errors to be weakly cross-correlated and heteroskedastic and (iii) weak correlation between the common factors and the errors. As discussed [7], the principal components estimator remains consistent in the case of the relaxing assumption (ii), i.e. when the errors are generated by stationary, low-dimension autoregressive processes.

In their seminal articles from 2002, [4] demonstrate that if an approximate factor model were applied to the original variables, then the model can be consistently estimated using the (standard) principal components of the sample covariance matrix of the original variables. This approach is further supported by [18], who argues that the principal components estimator has two significant advantages: (i) it can be easily computed and (ii) under the normality assumption, it is (asymptotically) equivalent to the maximum likelihood estimator. The author also determines the rate of convergence for the common components and shows that, somewhat counterintuitively, it can be slower than the rate of convergence of the estimated factors and factor loadings. At the same time, [5] show that, under certain circumstances, the common factors can serve as inputs in a regression model used to construct (asymptotically efficient) forecasts. Alternatively, the forecast can be performed by simply projecting the data onto the space spanned by the common factors (static principal components).

The work of [2,3] represents a major breakthrough in the area of forecasting economic processes using factor models. Their idea is based on the same principle as Stock and Watson’s model [4], but it takes a totally different approach to estimating the common factors, which are now estimated using generalized principal components. In this model, called the generalized dynamic factor model (GDFM), the common factors are still linear combinations of the data, but, unlike Stock and Watson’s model [4], the observations are weighted according to the signal-to-noise ratio (more on that later). The measure used to evaluate the correlations between the original variables is the spectral density matrix. As such, this framework specifically incorporates a dynamic element in the construction of the model.

It is important to note that the GDFM approach has been applied in a significant number of other studies [19–29] either as a stand-alone exercise or as benchmark against which other models were assessed. While most the authors state clearly that the GDFM has a significant potential to improve forecasting accuracy, it cannot be declared a priori that the GDFM outperforms competing models under all testing scenarios. For example, [30] show that, under the particular circumstance of disaggregated data series, as is generally the case of economic variables, dynamic factor models do not improve the forecasting results compared with static models. To this end, the authors compare the outputs of (i) a simple Stock-Watson model based on 20 aggregated data series and (ii) a dynamic model that includes 140 disaggregated variables. In the case of the Romanian economy, only one GDFM exercise has been performed, to our knowledge [31].

Investigating the limitations of the GDFM, several authors have noted that the mixed data frequencies inherent in large-scale factor models can be exploited in order to improve the accuracy of forecasting. This is discussed at length in [12];[25];[32], for example, who propose formal methods of incorporating variables with mixed frequencies in multifactor models. The authors show that, under certain circumstances, including the most recent monthly data generally improves the quality of the models’ outputs; however, this is largely confined to nowcasts or very short-term forecasts, i.e. one quarter ahead. In the case of forecasting horizons longer than one quarter, however, the literature provides little evidence that the most recent data makes a significant difference in terms of forecasting performance. Another interesting point is made by [26] who argues that in the case of emerging economies it is quite challenging to compile large data panels in a reliable fashion and that the alleged benefits of considering a large number of predictors (at least 30 or 40) are likely outweighed by issues of data reliability and oversampling of variables of the same type that may increase the cross-correlation of specific shocks across the time series used in the model. In order to address this issue, [33] resort to a medium-sized data panel that has the advantage of incorporating a larger quantity of information compared with small-scale models while also overcoming the technical limitations of large panels. The authors estimate the common factors using a Kalman filter technique and their respective relationships with the economic variables in the model by means of transfer functions.

Other important contributions to the forecasting of macroeconomic processes using factor analysis have been made by [6] and [34–36] among others. [34] propose a new approach in which the common factors follow an autoregressive process; under certain circumstances, their model outperforms the FHLR implementation [36]. [35] propose a two-step approach in which an initial estimation is performed using principal components. This estimation is then refined via a Kalman smoother [35]. A very useful comparison between the three models is provided in [6].

## Theoretical models applied in the research

### The generalized dynamic factor model

A detailed analysis of the concepts discussed in this sub-section is provided by [2,3] and [37–40]. Let $X_{n}^{T}=\left\{x_{it},i=1,\ldots ,n;t=1,\ldots ,\;T\right\}$ be the vector of *n* relevant time series at times *t = 1*, … , *T*. The model further assumes that $X_{n}^{T}$ is a finite realization of a real-valued, n-dimensional stochastic process *X* = {*x*_*it*_, *i* ∈ ℕ, *t* ∈ ℤ}, where for any *n* ∈ ℕ, the process *x*_*n*_ = (*x*_1*t*_, *x*_2*t*_, …, *x*_*nt*_)′, *t* ∈ ℤ, *n* ∈ ℕ is weak form stationary, with mean 0_*n*_ and finite second order moments ${\text{Γ}}_{nk}=E\left(x_{nt}x_{nt-k}^{\prime }\right),k\in \mathbb{N}$.

The GDFM as discussed in [3] is based on the principle that each variable *x*_*it*_ can be decomposed into the sum of two unobservable, orthogonal components: a common component, *χ*_*it*_, and a specific (idiosyncratic) component, *ξ*_*it*_. This assumption translates into the following equation [38–39]:

$$x_{it}=\chi_{it}+\xi_{it}=b_{i}^{\prime }\left(L\right)u_{t}+\xi_{it},i\in \mathbb{N},t\in \mathbb{Z}$$ (1)

which can be rewritten in the following way:

$$x_{it}=b_{i1}\left(L\right)u_{1t}+b_{i2}\left(L\right)u_{2t}+\cdots +b_{iq}\left(L\right)u_{qt}$$ (2)

if we ignore the specific component. In (Eq 2):

1. *u*_*t*_ is a q x 1 vector of common factors (q < n), satisfying *Var*(*u*_*jt*_) = 1, *Cov*(*u*_*t*_,*u*_*t*-*k*_) = 0 and *Cov*(*u*_*jt*_,*u*_*st*-*k*_) = 0, ∀ *j* ≠ *s*, *t*, *k*. This implies that *u*_*t*_ is an orthonormal process;
2. *ξ*_*n*_ = (*ξ*_1*t*_, *ξ*_2*t*_, …, *ξ*_*nt*_}′ is a weak form stationary process which satisfies the following: *Cov*(*ξ*_*jt*_,*u*_*st*-*k*_) = 0, ∀ *j* ≠ *s*, *t*, *k*. This implies that the common and specific components are orthogonal;
3. *b*_*i*_(*L*) is a q x 1 vector of bilateral filters, where *L* denotes the lag operator and $b_{ij}\left(L\right)=\overset{\infty }{\underset{k=0}{\sum }}b_{ij,k}L^{k},i=1,\ldots ,n;j=1,\ldots ,q$ are the factor loadings.

We now turn our attention to the algorithm used to estimate the common and idiosyncratic components of the data. First, we must derive reliable estimators of the spectral density matrices of the common and idiosyncratic components (let ${\text{Σ}}_{n}^{\chi T}\left(\theta \right)$ and ${\text{Σ}}_{n}^{\xi T}\left(\theta \right)$ denote these two matrices, respectively). As discussed in [1], we start by calculating the sample covariance matrices at all lag *k*:

$${\text{Γ}}_{nk}^{T}=Cov\left(X_{n}^{T},X_{n-k}^{T}\right),k=0,1,\ldots ,M$$ (3)

At this point a decision has to be made regarding the optimal number of lags (*M*). The authors suggest that the following approximation can be used:

$$M=\left[T^{1/3}\right]+1$$ (4)

For the purpose of estimating the spectral density matrix of the multivariate time process $X_{n}^{T}$, we resort to a discrete Fourier transform of the matrix sequence ${\text{Γ}}_{n,-M}^{T},\ldots ,{\text{Γ}}_{n,0}^{T},\ldots {\text{Γ}}_{n,M}^{T}$, where ${\text{Γ}}_{n,-k}^{T}={\text{Γ}}_{n,k}^{T}{}^{\prime }$. We shall have:

$${\text{Σ}}_{n}^{T}\left(\theta_{h}\right)=\frac{1}{2\pi }\sum_{k=-M}^{M}{\text{Γ}}_{nk}^{T}w_{k}e^{-ik\theta_{h}}$$ (5)

in which the frequencies *θ*_*h*_ are assumed to be distributed uniformly in the interval [0,2*π*]:

$$\theta_{h}=\frac{2\pi h}{2M+1},h=0,\ldots ,2M$$

The weights w_k_ are derived from the Bartlett lag window of size M:

$$w_{k}=1-\frac{\left|k\right|}{M+1},k=-M,\ldots 0,\ldots M$$

For all frequencies, *θ*_*h*_, *h* = 0, 1, …, 2*M* the eigenvalues $\lambda_{nj}^{T}\left(\theta_{h}\right),j=1,\ldots ,n$ of the spectral density matrix ${\text{Σ}}_{n}^{T}\left(\theta_{h}\right)$ and the corresponding eigenvectors $p_{nj}^{T}\left(\theta_{h}\right)$ are computed. Assume for convenience that the eigenvalues are sorted in descending order, which implies that

$$\lambda_{n1}^{T}\left(\theta_{h}\right)\geq \lambda_{n2}^{T}\left(\theta_{h}\right)\geq \ldots \geq \lambda_{nn}^{T}\left(\theta_{h}\right)$$

Let:

$$P_{n}^{T}\left(\theta_{h}\right)={\left(p_{n1}^{T}{\left(\theta_{h}\right)}^{\prime },\ldots ,p_{nq}^{T}{\left(\theta_{h}\right)}^{\prime }\right)}^{\prime },$$

denote the eigenvectors corresponding to the *q* largest eigenvalues of the spectral density matrix, and let:

$$Q_{n}^{T}\left(\theta_{h}\right)={\left(p_{n,q+1}^{T}{\left(\theta_{h}\right)}^{\prime },\ldots ,p_{nn}^{T}{\left(\theta_{h}\right)}^{\prime }\right)}^{\prime }$$

where denote the vector of the remaining *n–q* eigenvectors.

The two spectral density matrices can be expressed as the following matrix products [39–40]:

$${\text{Σ}}_{n}^{\chi T}\left(\theta_{h}\right)={\tilde{P}}_{n}^{T}\left(\theta_{h}\right)\left[\begin{array}{ccc} \lambda_{n1}^{T}\left(\theta_{h}\right) & \cdots & 0\\ \vdots & \ddots & \vdots \\ 0 & \cdots & \lambda_{nq}^{T}\left(\theta_{h}\right) \end{array}\right]P_{n}^{T}\left(\theta_{h}\right)$$ (6)

$${\text{Σ}}_{n}^{\xi T}\left(\theta_{h}\right)={\tilde{Q}}_{n}^{T}\left(\theta_{h}\right)\left[\begin{array}{ccc} \lambda_{n,q+1}^{T}\left(\theta_{h}\right) & \cdots & 0\\ \vdots & \ddots & \vdots \\ 0 & \cdots & \lambda_{nn}^{T}\left(\theta_{h}\right) \end{array}\right]Q_{n}^{T}\left(\theta_{h}\right)$$ (7)

in which the tilde means complex conjugation and vector transposition. We can now derive the covariance matrices of the common and idiosyncratic components using an inverse discrete Fourier transform:

$${\text{Γ}}_{nk}^{\chi T}=\frac{2\pi }{2M+1}\sum_{h=-M}^{M}\sum_{n}^{\chi T}\left(\theta_{h}\right)e^{i\theta_{h}k}$$ (8)

$${\text{Γ}}_{nk}^{\xi T}=\frac{2\pi }{2M+1}\sum_{h=-M}^{M}\sum_{n}^{\xi T}\left(\theta_{h}\right)e^{i\theta_{h}k}$$ (9)

It must be noted that the off-diagonal elements in the above estimations of ${\text{Γ}}_{nk}^{\xi T}$ are non-zero, because we allow the idiosyncratic factors to be correlated at all lags *k*. Discussing this issue, [3] argue that setting to zero all off-diagonal elements of matrix ${\text{Γ}}_{n0}^{\xi T}$ generally leads to improved estimations when the number *n* of time series is large when compared to the number of observations *T* (and this is the case of our model, as we shall discuss later). After computing the spectral density matrices, we must estimate the *r*-dimensional space of static factors. This is done by means of linear combinations of the original variables (the generalized principal components), as follows:

$$W_{nt}^{kT}=Z_{nk}^{T}X_{n}^{t}$$ (10)

in which the weights $Z_{nk}^{T},1\leq k\leq r$ solve the following optimization problem:

$$Z_{nk}^{T}=Arg{\text{max}}_{a}\alpha {\text{Γ}}_{n0}^{\chi T}\alpha^{\prime }$$ (11)

subject to the following restrictions:

$$\{\begin{array}{c} \alpha {\text{Γ}}_{n0}^{\xi T}\alpha^{\prime }=1\\ \alpha {\text{Γ}}_{n0}^{\xi T}Z_{nm}^{T\prime }=0 \end{array},1\leq m\leq k-1$$ (12)

It can be shown that the vectors $Z_{nj}^{T},j=1,\ldots ,n$ are the generalized eigenvectors corresponding to the generalized eigenvalues of the couple of the spectral densities $\left({\text{Γ}}_{n0}^{\chi T},{\text{Γ}}_{n0}^{\xi T}\right)$. For complete proof please see [2].

The common components are estimated using the following equations:

$$\chi_{it}^{nT}={\left({\text{Γ}}_{n0}^{\chi T}Z_{n}^{T}{\left({\tilde{Z}}_{n}^{T}{\text{Γ}}_{n0}^{T}Z_{n}^{T}\right)}^{-1}{\tilde{Z}}_{n}^{T}X_{n}^{t}\right)}_{i},t\leq T$$ (13)

where $Z_{n}^{T}={\left(Z_{n1}^{T}{}^{\prime },\ldots ,Z_{nr}^{T}{}^{\prime }\right)}^{\prime }$. These equations essentially provide us nowcasts of the common components. The *h*-step ahead forecasts of the common components are derived using the following expressions:

$$\chi_{i,T+h}^{nT}={\left({\text{Γ}}_{nh}^{\chi T}Z_{n}^{T}{\left({\tilde{Z}}_{n}^{T}{\text{Γ}}_{n0}^{T}Z_{n}^{T}\right)}^{-1}{\tilde{Z}}_{n}^{T}X_{n}^{T}\right)}_{i}$$ (14)

As previously discussed, the linear combinations $W_{nt}^{T}=\left(W_{nt}^{1T},\ldots ,W_{rt}^{nT}\right)$ are derived having in mind the objective of maximizing their variability for a given level of idiosyncratic variance, i.e. their signal-to-noise ratio is optimal. This implies that the model minimizes idiosyncratic risk, which can significantly improve the forecasting accuracy [19].

It is important to stress that for forecasting purposes we shall use only the common component and ignore the idiosyncratic component. While this may seem counterintuitive at first, we deem this a safe approach because studies are yet to prove that incorporating the idiosyncratic component in the GDFM produces better results [6]. That being said, if one wishes to include forecasts of the idiosyncratic component in the model, [3] argue that the idiosyncratic component can be reasonably forecast using univariate approaches or low dimension models such as vector autoregressions. However, it should be noted that this approach works best under the assumption of zero or mild cross-correlation between the idiosyncratic components.

### Stock and Watson model [4]

In their seminal paper [4], Stock and Watson model (SW) propose an approach that is similar to the GDFM model discussed in the previous section in several ways. One chief difference between the two models lies in the fact that the SW model tracks the comovoments in the original data using simple (static) covariances, and not the spectral density analysis described previously. The authors show that an approximate factor model can be reliably estimated by projecting the data onto the space spanned by the static principal components.

Let us consider the following expression of the sample covariance matrix at lag *l*:

$$\hat{\Gamma }\left(l\right)=\frac{1}{T-l}\sum_{i=l+1}^{t}Y_{i}Y_{i-l}^{\prime }$$ (15)

where Y denotes the vector of original data (here in standardized form), and $\hat{\Gamma }\left(0\right)$ represents the sample covariance matrix of the data. Define $\hat{\text{M}}$ as the diagonal matrix of the first *r* eigenvalues of $\hat{\Gamma }\left(0\right)$, sorted in descending order $\left({\hat{m}}_{1}\geq {\hat{m}}_{2}\geq \cdots \geq {\hat{m}}_{r}\right)$ and $\hat{S_{j}}$ as the corresponding eigenvectors, as follows

$$\hat{S}={\left(\begin{array}{ccc} {\hat{S}}_{1}^{\prime } & \ldots & {\hat{S}}_{r}^{\prime } \end{array}\right)}^{\prime }$$ (16)

The SW model produces nowcasts and forecasts of the relevant variable by projecting the original data onto the space spanned by the first *r* principal components, as follows:

$${\hat{\chi }}_{t}=\left[\hat{\Gamma }(0)\hat{S}\prime {\left(\hat{S}\hat{\Gamma }(0)\hat{S}\prime \right)}^{-1}\right]\left[\hat{S}Y_{t}\right]=\hat{S}\prime \hat{S}Y_{t}$$ (17)

$${\hat{\chi }}_{t+h|t}=\left[\hat{\Gamma }\left(h\right){\hat{S}}^{\prime }{\left(\hat{S}\hat{\Gamma }\left(0\right){\hat{S}}^{\prime }\right)}^{-1}\right]\left[\hat{S}Y_{t}\right]$$ (18)

where $\hat{\chi }$ denotes the common component. It is inherent in this model that the nowcasts and the forecasts are estimated based on the common component alone, i.e. the idiosyncratic component is ignored. This is identical to the GDFM discussed previously.

Comparing the SW model to their own GDFM, [3] highlight two main differences between these two approaches:

1. Because the SW approach is based on traditional covariance analysis, the model only incorporates the information included in $\hat{\Gamma }\left(\text{h}\right)$, where *h* is the lag of interest. In contrast to the SW model, the GDFM exploits the information contained in the entire series of sample covariances, $\hat{\Gamma }\left(\text{h}\right)$, i.e. both lagged and contemporaneous This ensures that the GDFM incorporates all relevant information to the maximum extent possible, because it accounts for all the dynamic cross-correlations between economic and non-economic variables that may be asynchronous.
2. In the GDFM, the spectral density matrices are used to produce estimates of the covariance matrices, which are then used to derive the optimal weights to be applied to the original data in order to calculate nowcasts/forecasts of the relevant variable(s). This is achieved by using the generalized principal components analysis, whereas the SW model is estimated using traditional principal components analysis. In other words, the factor space is approximated by the generalized principal components, as opposed to SW’s standard principal components.

Before moving on to the specifics of model design and applications to forecasting real GDP, we want to highlight some important aspects related to the statistical relevance and consistency of estimators in the GDFM and SW frameworks. While the formal proof is outside the scope of this paper, we believe it is important to the understanding of the relative performance of the models.

As discussed to some extent in [3], both the GDFM and the SW predictors are consistent, i.e. they converge in probability to the population optimal predictor. However, aside from the two differences that we mentioned previously, there is another primary reason to expect better performance from the GDFM: it exploits the correlations between the common and idiosyncratic components. The model maximizes the signal-to-noise ratio, i.e. it operates with time-varying factor loadings in the sense that the higher the idosyncratic component of a certain variable is, the lower the weight it is assigned. This relationship is dynamically reassessed with each new set of observations that becomes available.

While these two arguments apparently provide strong support in favour of choosing the GDFM over the SW model, it is all but impossible to provide a formal demonstration in this respect [2]. This conclusion is backed by certain studies which show that the GDFM does not necessarily outperform the SW model (e.g., [36] and [41]).

## Forecasting Romania’s real GDP

In this section, we first focus on constructing a GDFM to be used in the process of forecasting Romania’s real GDP. Our model is based on the previously described dynamic methodology and incorporates 86 economic and non-economic time series that are linked to economic output. We have gathered monthly and quarterly data spanning the period from Q1 2005 to Q4 2016 (for a total of 48 quarters) and we have not included time series for which values are unavailable at certain dates. The eligibility criterion for the variables is a correlation coefficient between the candidate variable and real output of no less than 0.3 in absolute value; however, other variables, which do not fulfil this criterion, have been selected due to their influence on real GDP. The variables have been adjusted for seasonality and working days and have been further been adjusted by calculating the quarterly percentage rates of change in order to obtain stationary inputs for the model. (However, there are some variables for which different adjustments have been performed. Please see S1 Table for further details). The primary data sources are [42–45]. The full dataset is included in S1 Table, along with the transformations applied to each variable in the model.

The first challenging issue in estimating the model is selecting the appropriate number of common factors *q*. The first (and often the most obvious choice) is to apply the so-called “scree-test”, as described in [46]. The main idea of this method is to analyze the graph of the eigenvalues (sorted in descending order) and identify the point in the graph where the line begins to level off; this should give an estimation of the number of factors to be included in the model. Another rule of thumb is the Kaiser-Guttman criterion that suggests a number of common factors equal to the number of eigenvalues (e.g., of the correlation matrix) greater than one. However, as discussed in [7], these methods are often fairly subjective and lack solid scientific foundation.

Other, more objective and scientific criteria can be used in the case of complex macroeconomic models that include a large number of variables, such as the metrics provided by [17]; [47–48]. However, the maximization of such information criteria does not warrant optimum performance for the model and, at the same time, overestimating the number of common factors does not produce significantly distorted nowcasts and forecasts (for a complete discussion on the topic see [2]).

As previously discussed, contemporary and future projections of real GDP shall be estimated based on the common component alone using the formula below:

$${\hat{y}}_{t+h|t}=k{\hat{\chi }}_{t+h|t}\sigma +\mu$$ (19)

in which *h* is the number of periods in the future for which we perform the forecast and *k* is a coefficient used to upscale the standard deviation of real GDP. This is necessary because the variable we are modelling is standardized real GDP, i.e. with mean equal to zero and variance equal to one. The common component is forecast using the GDFM described in the previous section of the paper.

The existing literature provides few, if any, indications as to the choice of *k*. In our view there can be two approaches here:

1. Set *k* equal to the value that minimizes some measure of error, i.e. the RMSE of the forecasts over some period. This approach has the disadvantage that it could lead to values of *k* that are hard to justify from an economic perspective. At the same time, as illustrated by [19], it could lead to high values (i.e., greater than two) of the scaling coefficients for which no objective explanation can be provided.
2. Set *k* equal to a value that compensates for the part of the information in the original dataset that is lost by selecting a number of common factors that is substantially less than the number of the original time series.

Our GDFM includes 86 explanatory variables, three common components and a maximum of four lags (which leads us to a number of 15 ‘static’ factors). In our view this choice represents a good trade-off, because we preserve approximately 72% of the information contained in the original dataset. Even though GDFMs usually work with far less explanatory power [7] and with only one or two common components [21], it is difficult to reconcile such a heterogeneous dataset with the objective of minimizing forecasting errors and one possible (and plausible choice) is to increase the number of factors.

The exercise of forecasting real GDP will be performed for a sixteen-quarter horizon, i.e. over the period Q1 2014 –Q4 2017, and it will be done in several steps:

(i) three full year forecasts (2014–2016), based on the full dataset up to the last quarter of the previous year (e.g., the forecast for 2016 will consist of four individual quarterly GDP forecasts with 1-, 2-, 3- and 4-steps ahead starting from Q4 2015). Using this approach, we shall also estimate quarterly real GDP growth rates for 2017;
(ii) twelve 1-step ahead quarterly forecasts, where the latest available information is at the end of the previous quarter (this is essentially a nowcast);
(iii) twelve 2-step ahead quarterly forecasts, where the latest available information is at the end of Q-2;
(iv) twelve 3-step ahead quarterly forecasts, where the latest available information is at the end of Q-3;
(v) twelve 4-step ahead quarterly forecasts, where the latest available information is at the end of Q-4.

Where Q denotes the quarter for which real GDP is forecast. The forecasts described at points (ii) to (v) described above cover the period from Q1 2014 to Q4 2016.

The performance of the model shall be appraised using the root mean square error (RMSE) indicator, defined as follows:

$$RMSE=\sqrt{\frac{1}{H}\sum_{t=1}^{H}{\left(\hat{Y_{t}}-Y_{t}\right)}^{2}}$$ (20)

**Fig 1:**
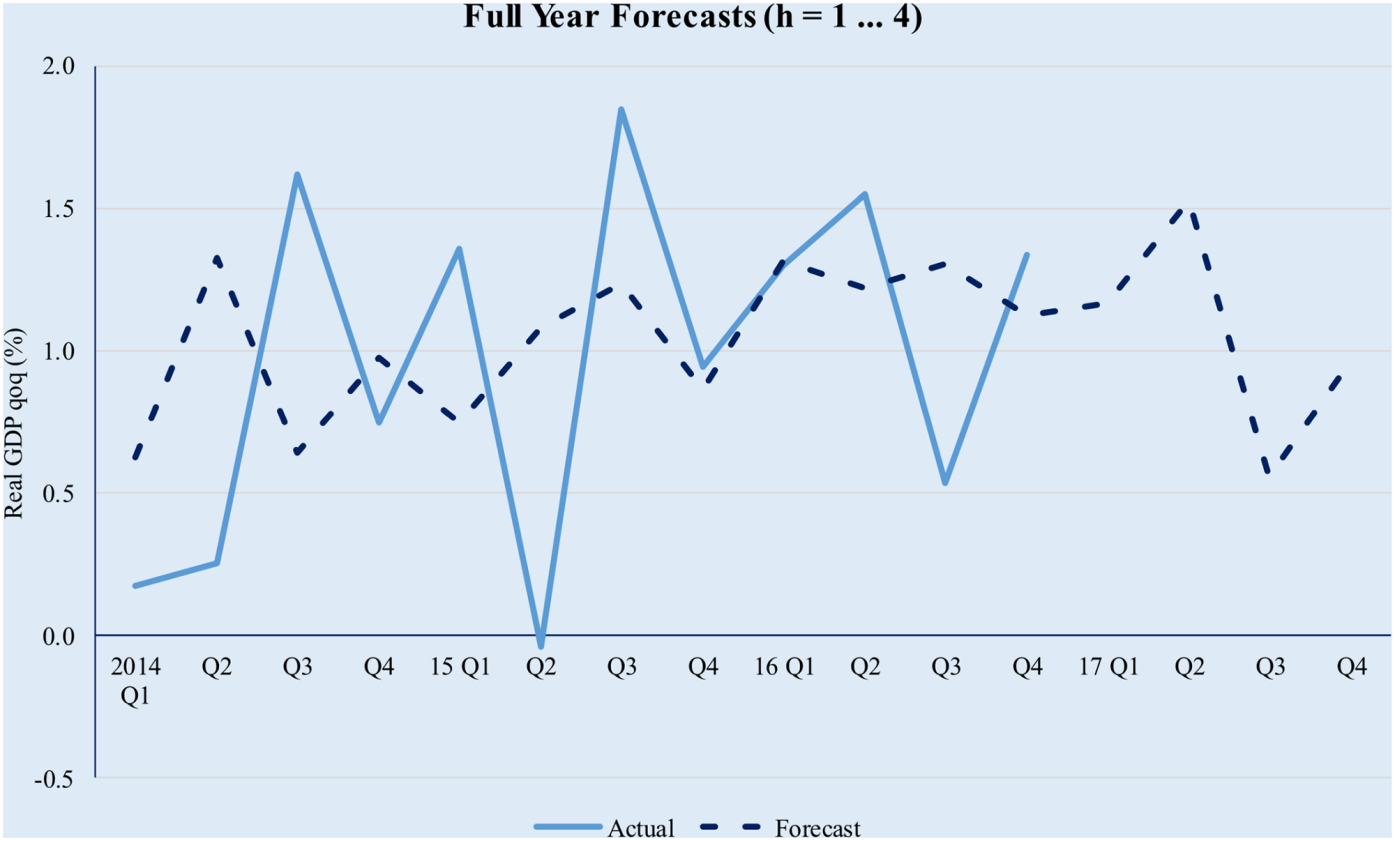
Quarterly GDP forecasts in a GDFM framework (full year estimation). Source data from [42].

**Fig 2:**
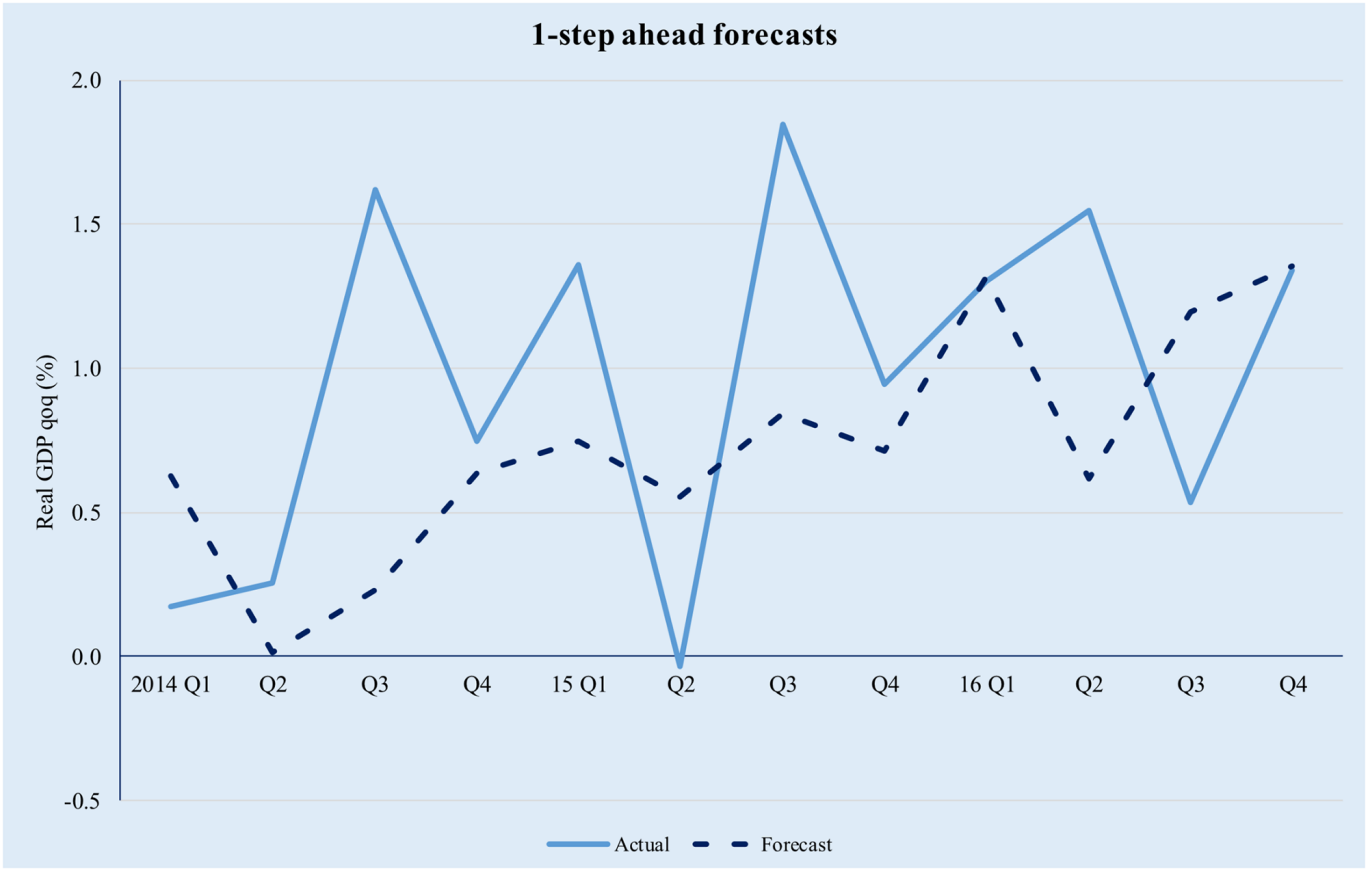
Quarterly GDP forecasts in a GDFM framework (1-step ahead). Source data from [42].

**Fig 3:**
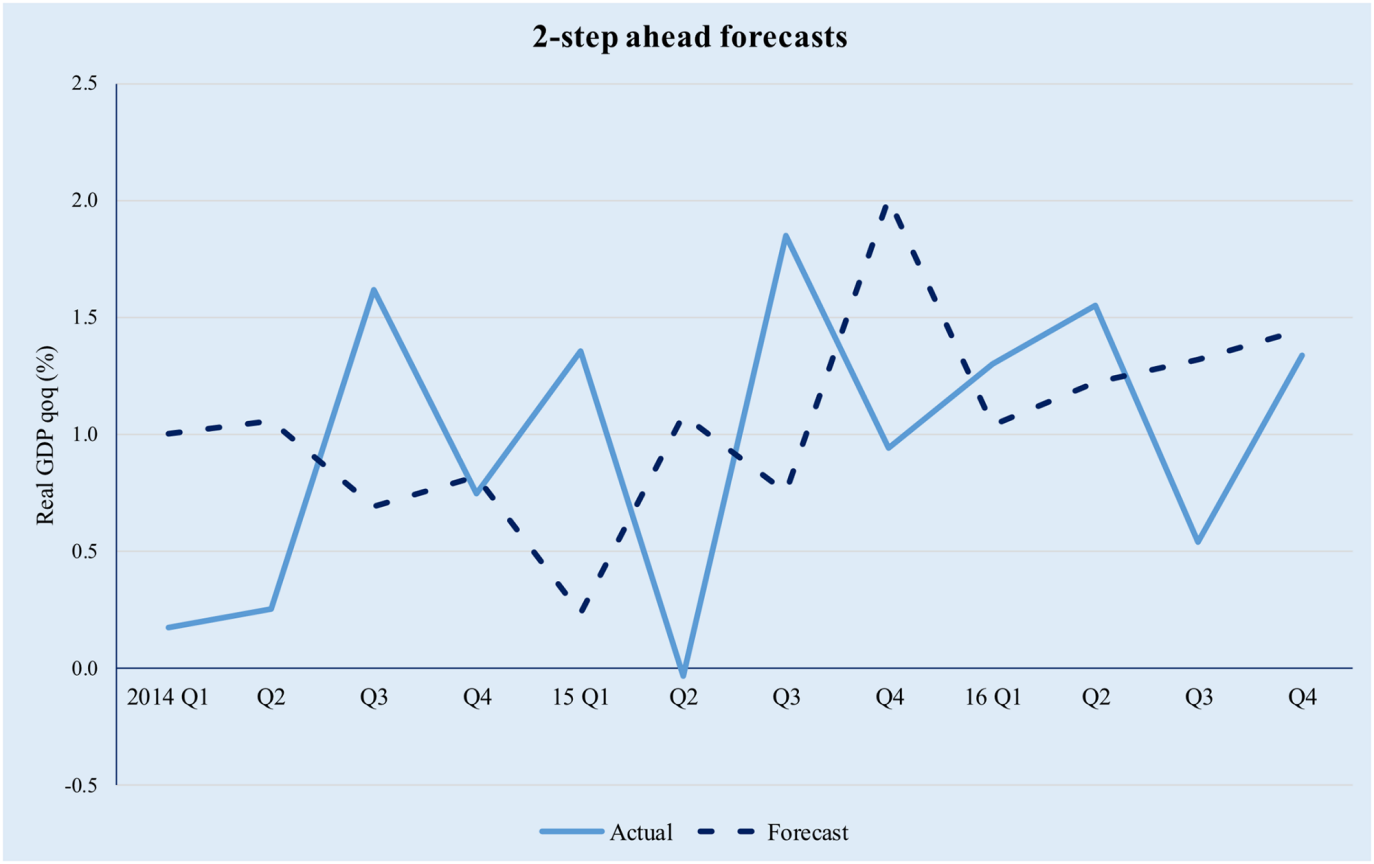
Quarterly GDP forecasts in a GDFM framework (2-step ahead). Source data from [42].

**Fig 4:**
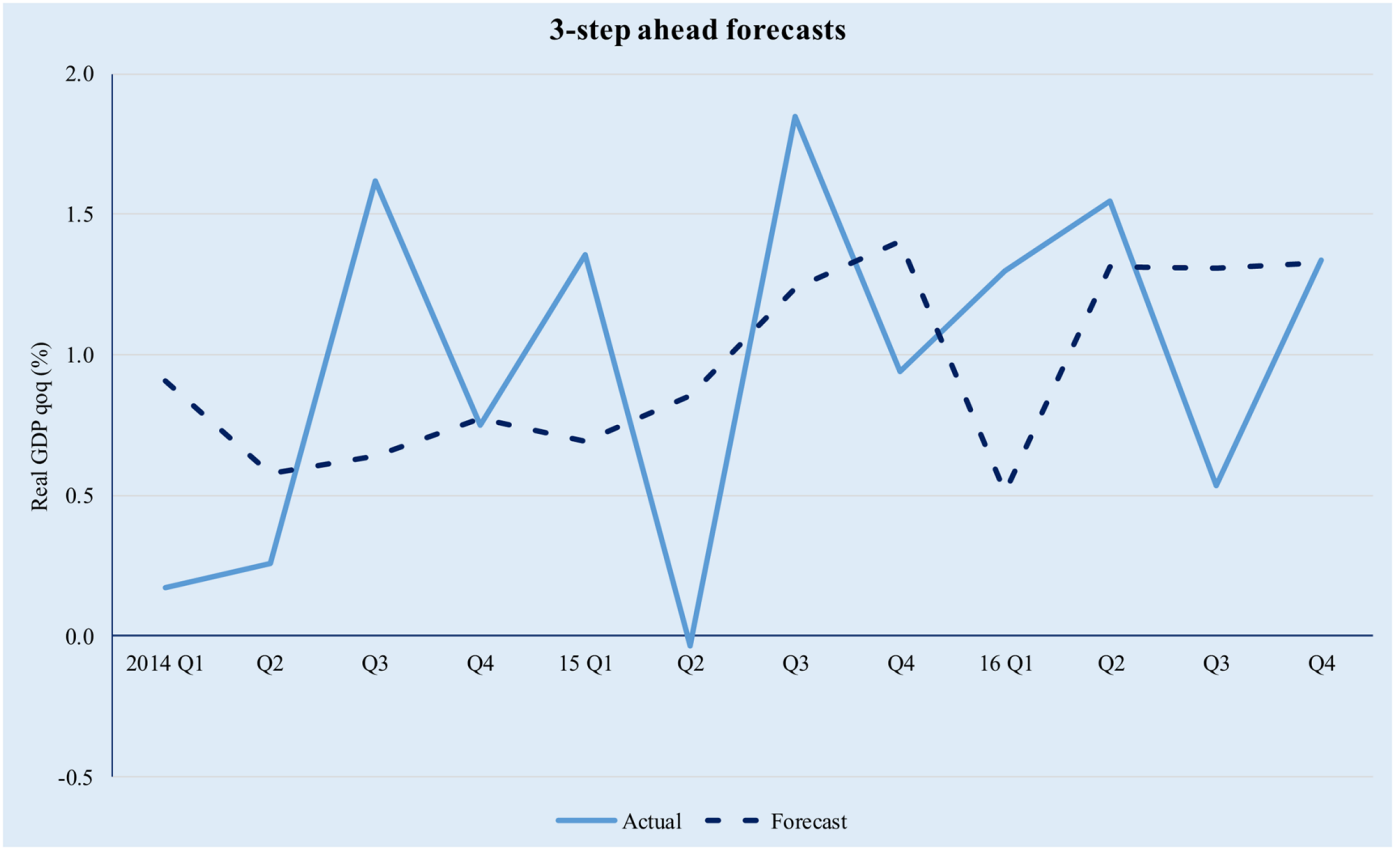
Quarterly GDP forecasts in a GDFM framework (3-step ahead). Source data from [42].

**Fig 5:**
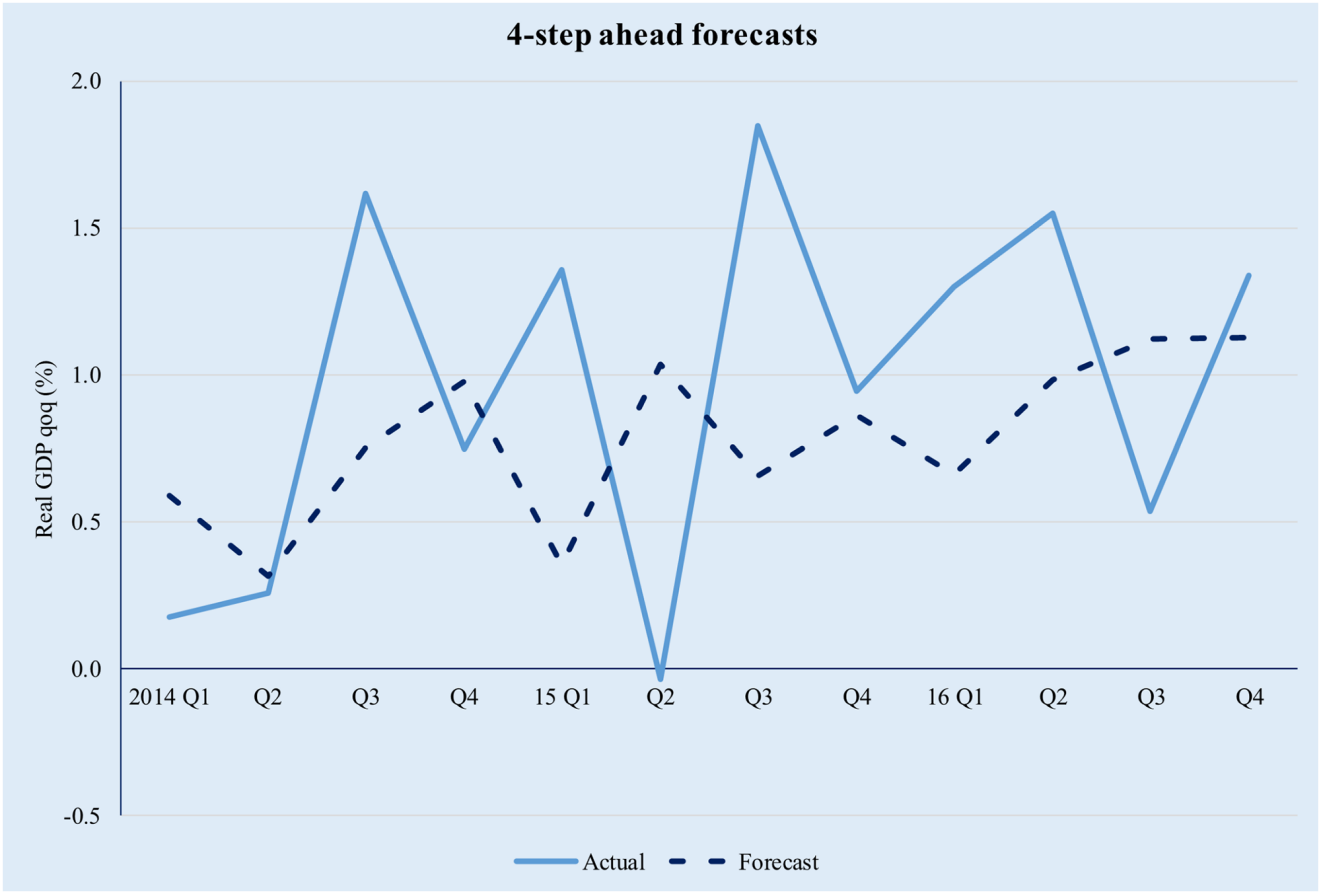
Quarterly GDP forecasts in a GDFM framework (4-step ahead). Source data from [42].

The model has been implemented using the Matlab^™^ R2016b software suite, based on the code uploaded by professor M. Forni (as described on http://morgana.unimore.it/forni_mario/matlab.htm) and publicly available at the time this paper was written. The results are presented in Figs 1–5 below.

The model tracks the evolution of real GDP quite well, albeit higher-order forecasts are clearly affected by the compounding of errors. The latter is especially true in the context of significant volatility in the Romanian macroeconomic environment and while in theory forecasting performance could be improved by modelling the idiosyncratic component; in practice it is difficult to achieve forecasting improvement by trying to explicitly incorporate the idiosyncratic factors. This is because (i) we only use 12 years of data (i.e., 48 quarters) and as a result the training set is limited and (ii) the observed volatility of the idiosyncratic component is too high for simple, univariate modelling techniques to yield meaningful results.

However, it is not at all clear that incorporating the specific component will lead to improved forecasts, not least because of the inherent challenge in modelling the process. In this context, we refrain from performing such an analysis as it will likely induce significant volatility in the GDP forecasts and thus undermine the reliability of the GDFM. Nevertheless, as discussed by certain authors (see, for example, the analysis in [31]), there are various ways of enhancing the output of the GDFM, i.e. by estimating confidence intervals and by incorporating expert hypotheses as to the future state of the economy. One possible way to build confidence intervals is to use the bootstrapping of errors technique and evaluate the relevant interquartile range.

Another way to enhance the GDP forecasts would be to estimate the mean of the resulting empirical distribution and to add it to the model results as a margin of conservatism. The quality of the forecasts is demonstrated by the relatively low levels of the corresponding RMSE indicators, as shown in Table 1:

**Table 1:** RMSE of h-step ahead forecasts. Source: authors ‘own calculations.

Real GDP Forecast	RMSE (%)
1-step ahead	0.65
2-step ahead	0.79
3-step ahead	0.86
4-step ahead	1.18

**Fig 6:**
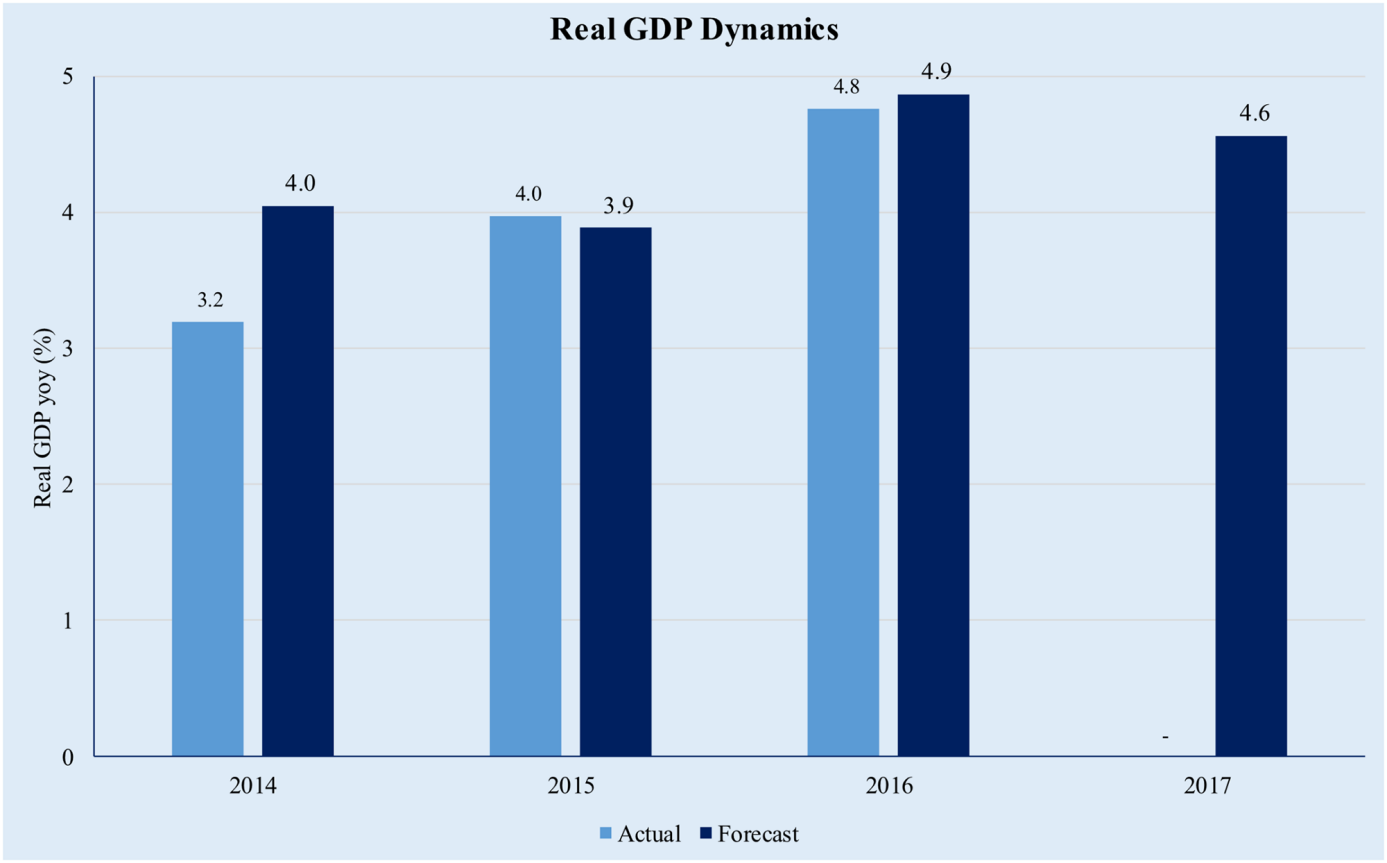
Yearly GDP forecasts in a GDFM framework. Source data from [42].

If we analyze the full year forecasts we see that the estimated yearly growth rates are pretty close to the actual outcomes as illustrated in Fig 6 below:

As regards the forecast for 2017, our current estimation is a growth rate of 4.6%, which is roughly in line with the expected real growth of 4.4% estimated by the European Commission in its Winter 2017 Economic Forecast and the growth rate of 4.2% estimated by the International Monetary Fund [49];[50]. In order to refine further the forecast, we want to assess the contribution of the variables included in the model to the change in real GDP. In order to evaluate the contributions, we have grouped the time series in eight broad categories, as follows:

- core variables (mainly GDP and sectoral output components)
- price indicators (inflation rates)
- financial and monetary variables (monetary aggregates, interest rates, nominal and real exchange rates, market indicators)
- balance of payments and net international investment position (mainly variables related to current account, foreign direct investment, reserve assets and government debt)
- labour market variables (employment, wages etc.)
- activity rates (mainly turnover, productivity)
- survey indicators (economic sentiment, industrial confidence, consumer confidence etc.)
- international variables (e.g., spot and forward oil prices, euro area prices, output, investment and foreign trade, US output and prices)

**Fig 7:**
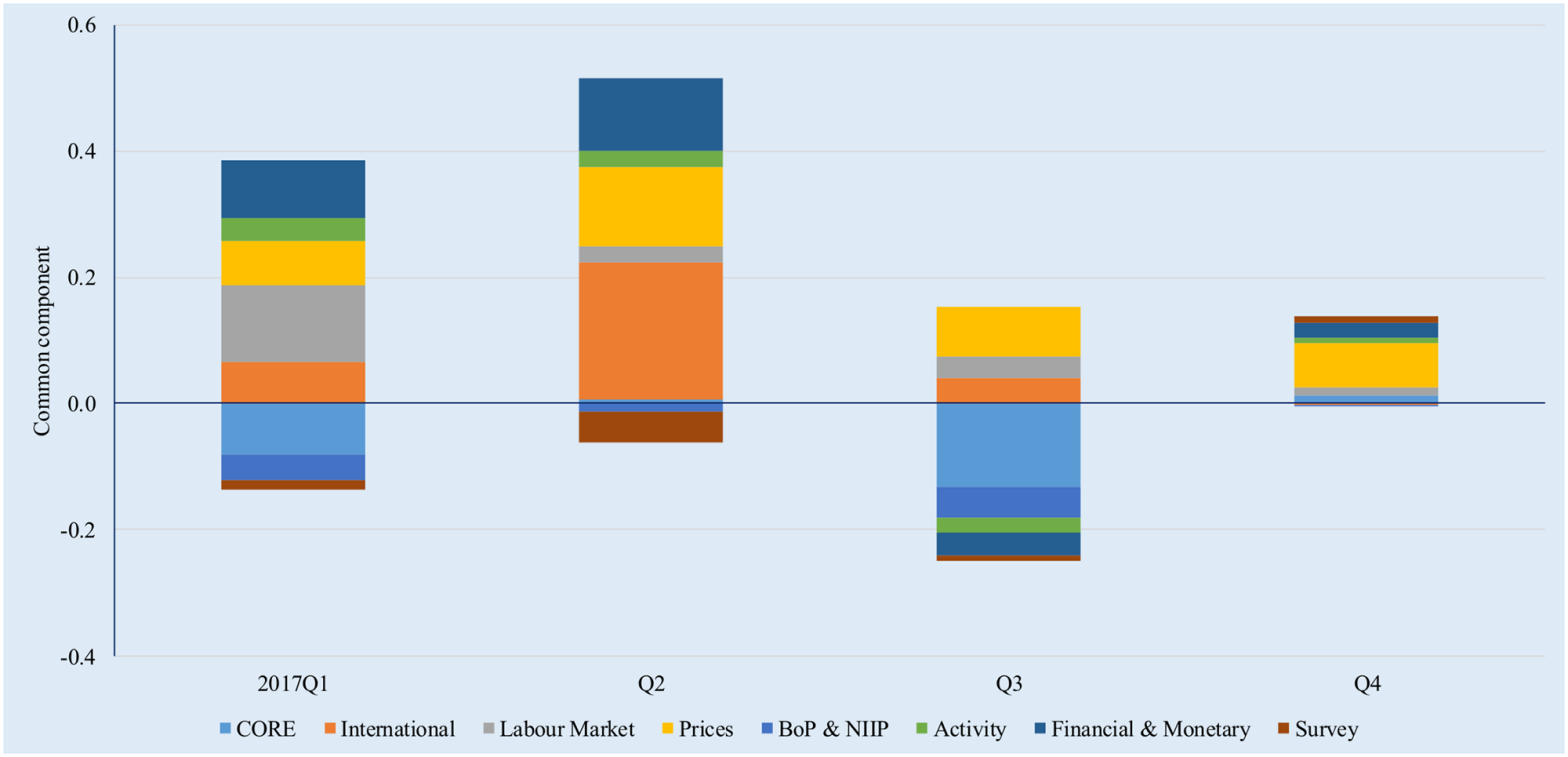
Drivers of GDP dynamics, 2014–2016. Source: authors’ own calculations.

**Fig 8:**
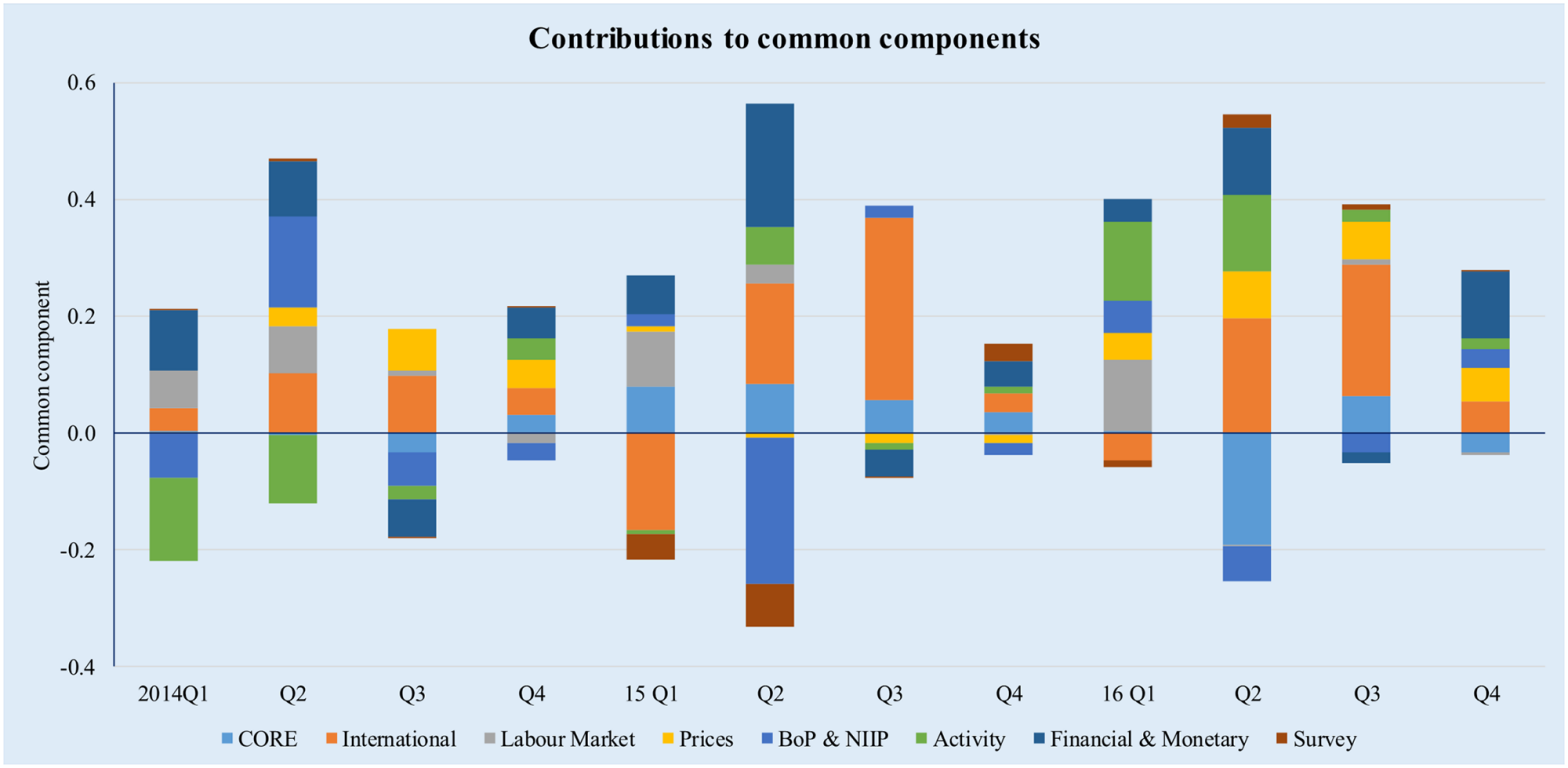
Drivers of GDP dynamics, 2017. Source: authors’ own calculations.

The correspondence between the variables included in the GDFM and these eight broad categories is included in S1 Table. The Figs 7 and 8 below provide an illustration of the factor contributions to the common components estimated using the GDFM.

It is interesting to note that the contributions predicted by the GDFM are largely in line with the actual economic developments in Romania over the past three years. Economic growth was mostly supported by surging consumer demand helped by expansionary fiscal and wage policies, i.e. VAT and other tax reductions, as well as significant increases in minimum wages and public sector compensation. While resilient domestic demand has certainly helped improve the economic outlook, economic growth has not been accompanied by gains in productivity and economic policies aimed at stimulating aggregate supply, which in turn raises question marks as to the sustainability of economic growth. At the same time, the model correctly captures the weak dynamics of investment and the negative contributions of net exports to GDP growth, which are reflected in the projected negative contributions of the CORE variables to the common components in 2016 and 2017.

Economic growth was also supported by the accommodative monetary and financial conditions, with record low interest rates benefiting both households and companies. That being said, lending growth remains subdued, mostly because companies are reluctant to take on leverage that could unlock significant growth opportunities if managed correctly. According to recent central bank estimates, the corporate loan stock could double and still remain sustainable while significantly lowering the overall cost of capital for Romanian companies [51];[52].

The activity indicators have had a positive contribution to economic growth over most of the three year period. However, this development does not tell the full story because indices of turnover and production generally increase as the economic activity increases, i.e. due to a surge in domestic demand; when interpreting the results, we must also consider the weak gains in productivity and increase in unit labour costs that hamper Romania’s competitiveness and may lead in time to reduced domestic and foreign demand.

The decline in oil prices and the domestic deflationary pressures generated by the cut in taxes on consumption have also been supportive of GDP growth, however this is expected to change as the basis effect disappears and inflationary pressures start to mount because of rapidly increasing labour costs. Absent a shift in the current procyclical stance of the fiscal and wage policies, a rapid increase in price is to be expected–we foresee headline inflation exceeding the central bank’s target interval of 2.5 +/- 1% in the second half of 2018. This poses further risk to the sustainability of economic growth because the central bank might find itself in the position to perform the delicate balancing act between tightening monetary conditions to contain inflation and maintaining a policy stance that is supportive of sustainable growth. Fiscal policy will be instrumental here, otherwise there is a clear risk of suboptimal policy mix.

The labour market [53–55] has also contributed positively to economic growth, with unemployment reaching record low levels and rapidly increasing wages that have fed domestic demand for consumption. While low joblessness is a good sign, Romania still exhibits substantial regional disparities, very low workforce mobility and a lack of highly trained workers that are required especially in the high-tech sector. Structural reforms in this area should address improvements in labour mobility, including the provision of financial incentives, as well as continuous education and training in response to the continuous changes in labour market conditions.

Starting in 2015, the improving international economic context has positively influenced the Romanian economy, due to the rebound in economic sentiment in Romania’s main trading partners. Nevertheless, significant external risks persist, mostly in connection to the current situation in Europe, the yet unclear economic policies of the new American administration, as well as the divergence between the monetary policies of the ECB and the Fed. At the same time, survey indicators have had a limited contribution to GDP forecasts–unsurprising, as their relevance is limited to nowcasts of economic output, in line with the findings of [24].

In order to assess the relative performance of the GDFM, we have also developed two SW models using (i) the full dataset as in the case of the GDFM and (ii) a reduced dataset consisting of 65 variables (please refer to S2 Table for the complete specification of second SW model). The predictions are carried out in the same way as with the GDFM, i.e. using only the common components. The number of common factors has been selected so as to ensure an adequate degree or preservation of information while also significantly reducing the dimensionality of the original space. The number of principal components has been selected so as to ensure comparable degrees of information retention with the GDFM. The details are given in Table 2 below.

**Table 2:** Two SW models. Source: authors’ own calculations.

Model	# of Variables	# of Common Components	Information Retention
SW_1_	86	10	71%
SW_2_	65	11	78%

Note: Information Retention is computed as the average of information preservation over the entire time horizon and across all lags considered, i.e. Q1 2005 –Q4 2016 and 1 to 4 lags.

Somewhat unsurprisingly, choosing either of the two SW models over the GDFM leads to significant increases in the forecasting errors, as shown by the RMSE (see Table 3). This happens even in the case of very short-term forecasts, i.e. 1- and 2-quarter ahead, which leads to the empirical conclusion that the SW model fails to properly account for the correlation between the predictors.

**Table 3:** Increase in RMSE vs. GDFM (%), # of quarters ahead (forecast). Source: authors’ own calculations.

Model	1	2	3	4
**SW**_**1**_	41%	43%	86%	81%
**SW**_**2**_	47%	63%	105%	70%

This is the fundamental flaw of static principal components analysis that is addressed by the GDFM by exploiting the dynamic correlations between the variables, i.e. using spectral density analysis. While it may be argued that in the case of smaller datasets the SW framework will likely perform better, it is obvious that the model has severe limitations in what concerns the accommodation of large numbers of variables that contain information that is potentially useful in forecasting real GDP.

We now turn our attention to assessing the relative performance of the three models. While considerable research has been devoted to the rigorous comparison between the two models from a theoretical standpoint, in practice there is little to be done besides comparing some measure of error across model specifications. In this respect, we have opted for the root-mean-square error criterion (RMSE), which has gained significant popularity, with many researchers (see, for example, [19], [20], [23] and [25]). The results of the comparison are given in Table 3 below.

**Fig 9:**
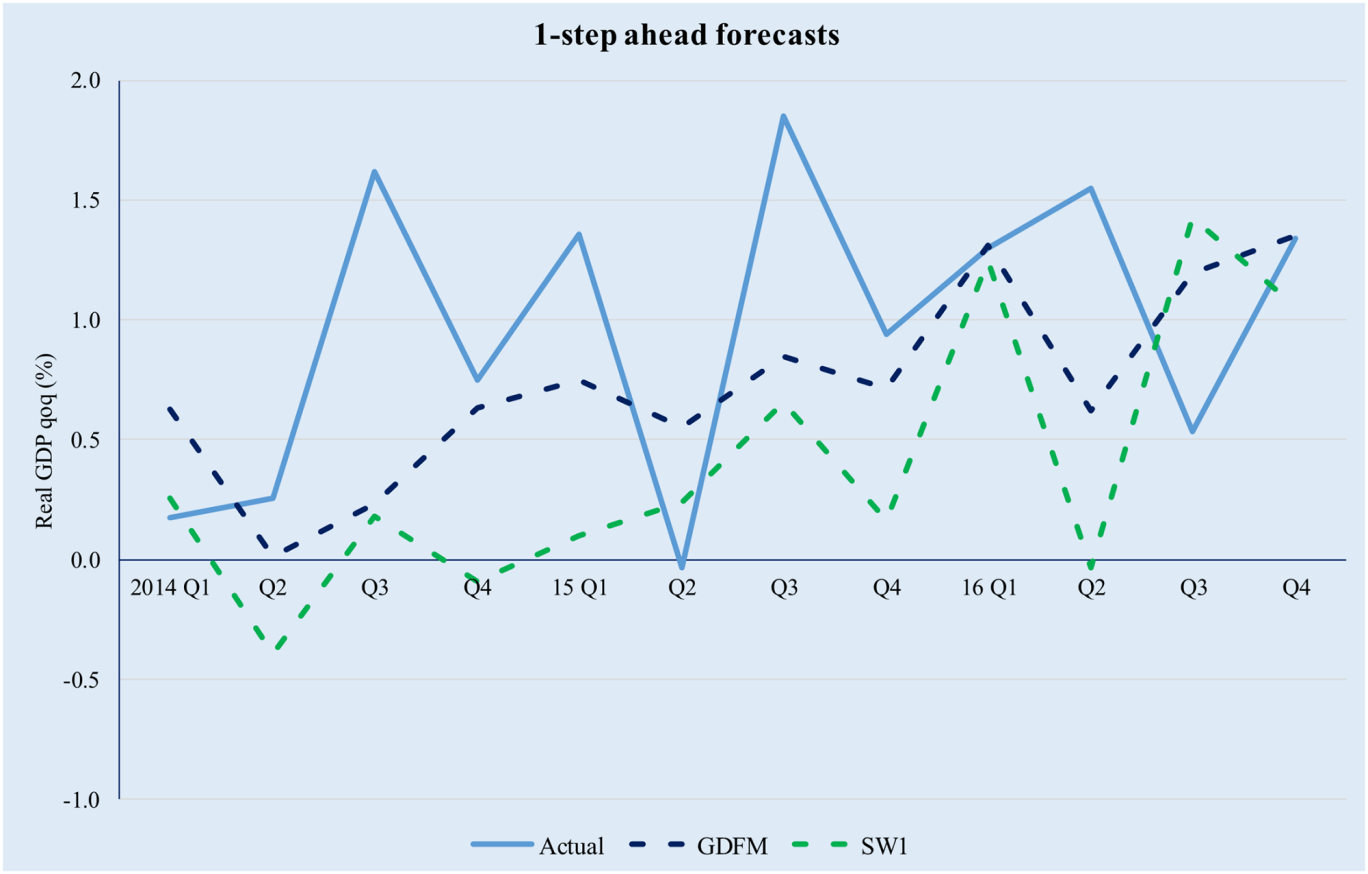
GDFM vs SW, 1-step ahead forecasts. Source: authors’ own calculations.

**Fig 10:**
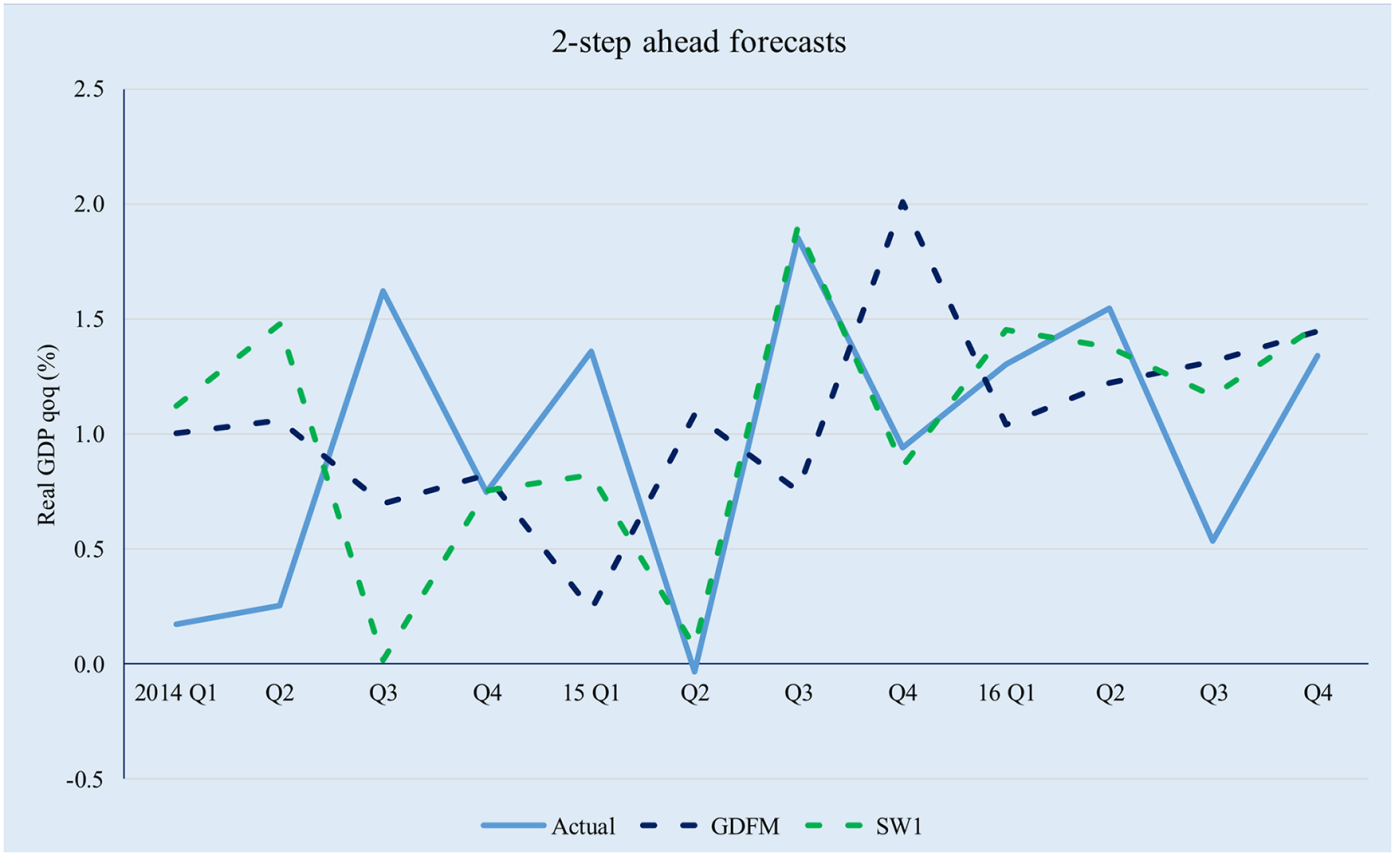
GDFM vs SW, 2-step ahead forecasts. Source: authors’ own calculations.

**Fig 11:**
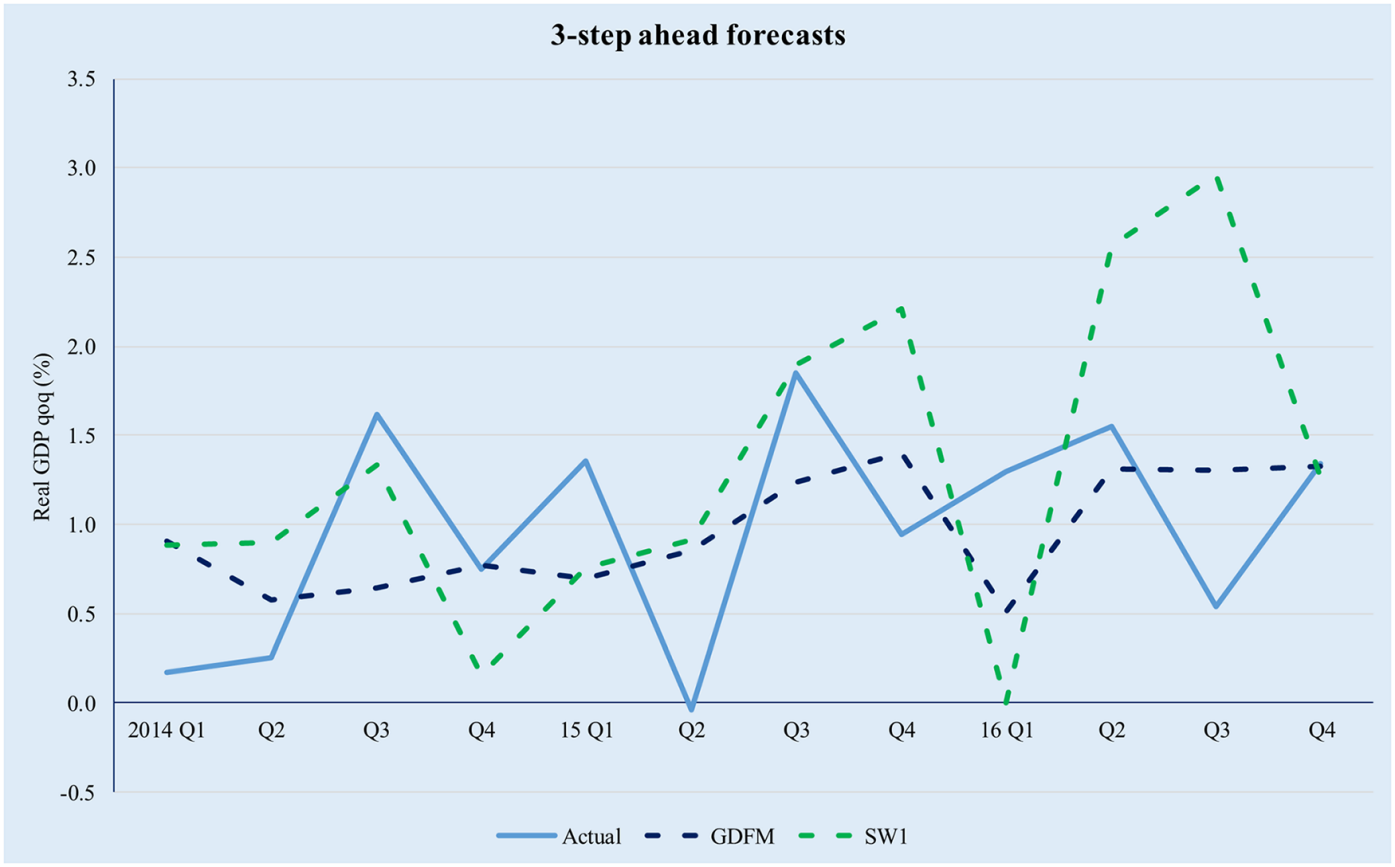
GDFM vs SW, 3-step ahead forecasts. Source: authors’ own calculations.

**Fig 12:**
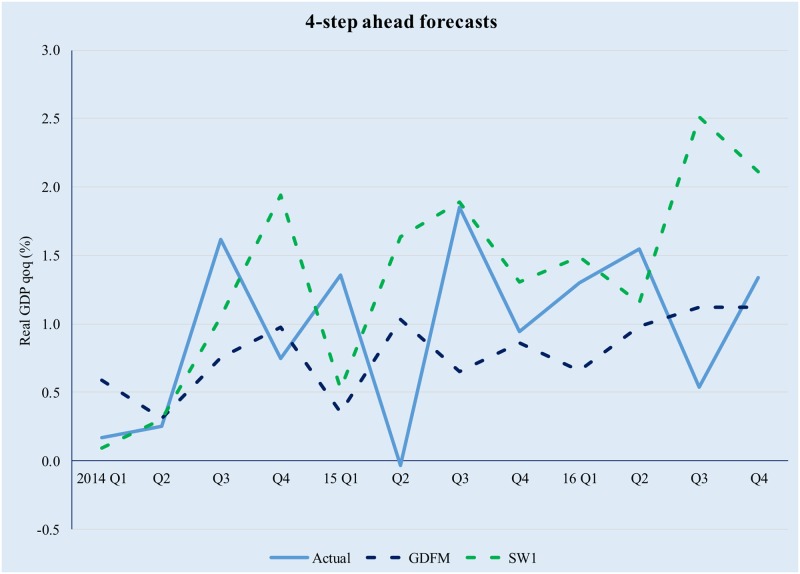
GDFM vs SW, 4-step ahead forecasts. Source: authors ‘own calculations.

It is interesting to note that, even though the three competing models preserve comparable amounts of information, the predictive power of the GDFM is vastly superior to the two SW models, due to the way the GDFM works, i.e. by maximizing the signal-to-noise ratio. This enables it to achieve significant gains compared with the static models, with the latter also being somewhat disconnected to the evolution of actual real GDP. Figs 9–12 provide a relevant comparison of the forecasting accuracy of the GDFM and SW_1_ over 1- to 4- forecasting horizons. For the purpose of the present comparison we have chosen the SW1 model because it calibrated based on the same dataset as the GDFM

While it is clear that the SW approach has its own merit, it is also obvious that it struggles to deal with large data panels and to capture the complex relationships between economic variables. Given the large number of variables considered for our model, we consider the GDFM approach to be more appropriate and we recommend using the SW framework with smaller, balanced data sets, as many other studies have suggested.

Finally, we have also attempted to assess the relative performance of the GDFM against standard univariate models such as ARMA(p,q), with parameters *p* and *q* ranging from 1 to 4. As was to be expected, we haven’t managed to design a meaningful model of this class, with R-squared indicators generally less than 0.5. Intuitively, this happens because of the significant volatility in quarterly real GDP, with the sizable idiosyncratic shocks confusing the comparatively simple linear models.

## Conclusions

In this research, we demonstrate the relevance and usefulness of a dynamic factor model approach to forecasting real GDP growth. In this respect, we have developed a model that incorporates 86 explanatory variables which have been subsequently reduced to just three dynamic common factors that account for approximately 72% of the information contained in the original dataset. This model has been used to produce quarterly forecasts of real GDP, as well as to identify the key drivers of economic growth and to provide some policy recommendations.

Compared to traditional models (e.g., VAR, ARIMA, and even static principal components analysis), our model has the advantage of dynamically exploiting a large amount of information and, at the same time, being able to assess the contribution of each descriptive variable to the forecast growth. This is especially important in the current context of forecasters operating with huge amounts of data who need an objective quantitative criterion to evaluate the contribution of each time series to their forecasts, as well as to be able to quantify the impact of larger subsets of variables to economic growth.

Nevertheless, there is still room for improvement in the GDFM. Firstly, we do not take into account the idiosyncratic component, whose accurate modelling–difficult as it may be–might yield superior results to our approach. Secondly, we do not update our forecast based on subsequent data with different frequency, i.e. we do not resort to an unbalanced dataset approach as in other similar studies. However, as discussed, in this case the potential for improvement is confined to the very short-term. Lastly, the model could be expanded by building confidence intervals using the bootstrapping technique and/or incorporating expert opinions, as shown by [31]. We did not resort to this approach because (i) it increases the degree of subjectivity and (ii) we do not have sufficient observations in order to isolate a relevant training set to be used for error prediction and modelling. This is of great interest to economic policy makers because, unlike simple univariate techniques or methods based on bridge equations, it is possible to evaluate the impact of broad groups of variables and adjust the policy mix accordingly.

## Supporting information

S1 Table (DOCX)

S2 Table (DOCX)

## References

[1] Bernanke B; Boivin J; Monetary Policy in a Data-Rich Environment, Journal of Monetary Economics 2003; (50): 525–46.

[2] Forni M; Hallin M; Lippi M; Reichlin L. The Generalized Dynamic Factor Model: Identification and Estimation, The Review of Economics and Statistics 2000; 82(4): 540–54.

[3] Forni M; Hallin M; Lippi M; Reichlin L. The Generalized Dynamic Factor Model: one-sided estimation and forecasting, Journal of the American Statistical Association 2005; 100(471): 830–40.

[4] Stock J; Watson M. Forecasting using principal components from a large number of predictors, Journal of the American Statistical Association 2002; 460(97): 1167–79.

[5] Stock J; Watson M. Forecasting using principal components from a large number of predictors, Journal of Business and Economic Statistics 2002; 20(2): 147–62.

[6] Barhoumi K; Darné O; Ferrara L. Are Disaggregate Data Useful for Factor Analysis in Forecasting French GDP? DT 232/2009, Banque de France.

[7] Breitung J; Eickmeier S. Dynamic Factor Models Deutsche Bundesbank DP 38/2005.

[8] Geweke J. The Dynamic Factor Analysis of Economic Time Series Models In Aigner D and Goldberger A (editors) *Latent Variables in Socioeconomic Models* 365–383, Amsterdam, North-Holldand, 1977.

[9] Sargent Y; Sims C. Business Cycle Modelling Without Pretending to Have Too Much A Priori Economic Theory, WP 55/1977, Federal Reserve Bank of Minneapolis.

[10] Engle RF; Watson M.A One-Factor Multivariate Time Series Model of Metropolitan Wage Rates Journal of the American Statistical Association 1981; 76: 774–81.

[11] Quah D; Sargent TA Dynamic Index Model for Large Cross-Sections, DP 77/1992, Federal Reserve Bank of Minneapolis.

[12] Giannone D; Reichlin L; Small D. Nowcasting: The real-time information content of macroeconomic data, Journal of Monetary Economics 2008; 55: 665–76.

[13] Connor G; Korajczyk RA. Performance Measurement with the Arbitrage Pricing Theory: A New Framework for Analysis, Journal of Financial Economics 1986; 15: 373–94

[14] Connor G; Korajczyk RA Risk and Return in an Equilibrium APT: Application of a New Test Methodology, Journal of Financial Economics 1988; 21(2): 255–89.

[15] Forni M; Hallin M; Lippi M; Reichlin L. Coincident and leading indicators for the Euro area, Economic Journal 2001; 111(471): 62–85.

[16] Chamberlain G.; Rothschild M.Arbitrage, Factor Structure, And Mean-Variance Analysis on Large Asset Markets, Econometrica 1983; 51(5): 1281–1304.

[17] Bai J; Ng S. Determining the Number of Factors in Approximate Factor Models, Econometrica 2002; 70(1): 191–221.

[18] Bai J. Inferential Theory for Factor Models of Large Dimensions, Econometrica 2003; 71(1): 135–71.

[19] Rogleva P. Short-Term Forecasting of Bulgarian GDP Using a Generalized Dynamic Factor Model, DP/86/2011, Bulgarian National Bank.

[20] Kabundi A; Gupta R. A dynamic factor model for forecasting macroeconomic variables in South Africa, Journal of Economic Literature 2008, available at: http://hdl.handle.net/2263/7409 [Retrieved 11 April 2017].

[21] Van Nieuwenhuyze C. A Generalised Dynamic Factor Mode/l for the Belgian Economy. Useful Business Cycle Indicators and GDP Growth Forecasts WP 80/2006.

[22] Masten A; Glazar M.; Kusar J; Masten I.Forecasting Macroeconomic Variables in Slovenia Using Dynamic Factor Models IMAD WP 9/2008.

[23] Ajevskis V; Davidsons G. Dynamic Factor Models in Forecasting Latvia’s Gross Domestic Product WP 2/2008, Latvijas Banka.

[24] Liebermann J. Real-time forecasting in a data-rich environment MPRA Paper No. 39452, 2012.

[25] Porshakov A; Deryugina E; Ponomarenko A; Sinyakov A.Nowcasting and Short-Term Forecasting of Russian GDP with a Dynamic Factor Model WP 2/2015, Bank of Russia

[26] Tóth P. Nowcasting Slovak GDP by a Small Dynamic Factor Model, MPRA Paper No. 77245, 2017.

[27] Artis M; Banerjee A; Marcellino M. Factor forecasts for the UK”, Journal of Forecasting 2005 24(4): 279–98.

[28] Forni M; Hallin M; Lippi M; Reichlin L. Do financial variables help forecasting inflation and real activity in the Euro area?, Journal of Monetary Economics 2003 50(6): 1243–1255;

[29] Schneider M; Spitzer M. Forecasting Austrian GDP using the generalized dynamic factor model, September 2004, WP 89.

[30] Barhoumi K; Darné O; Ferrara L. Are Disaggregate Data Useful for Factor Analysis in Forecasting French GDP?, DT 232/2009, Banque de France

[31] Tănase A. Estimation and Forecast of GDP and GDP Components with the Dynamic Factor Model. Application for Romania, NBR, Occasional Papers No. 12/2015.

[32] Marcellino M; Porqueddu M; Venditti F. Short-Term GDP Forecasting with a Mixed Frequency Dynamic Factor Model with Stochastic Volatility Banca d’Italia, 2012

[33] Cuevas A; Quilis E. A factor analysis for the Spanish economy, Journal of the Spanish Economic Association, 2012; 3(3): 311–38

[34] Doz C; Giannone D; Reichlin L. A quasi maximum likelihood approach for large approximate dynamic factor models, CEPR Discussion Paper No. 5724/2006.

[35] Doz C; Giannone D; Reichlin L. A two-step estimator for large approximate dynamic factor models based on Kalman filtering, CEPR Discussion Paper No. 6043/2007.

[36] Schumacher C. Forecasting German GDP Using Alternative Factor Models Based on Large Datasets, Bundesbank DP 24/2005.

[37] Anderson TW. An Introduction to Multivariate Statistical Analysis, Wiley, 1984.

[38] Brockwell P J; Davis RA. Time Series: Theory and Methods, 2^nd^ Edition, Springer, 2009.

[39] Fuller WA. Introduction to Statistical Time Series 2^nd^ Edition, Wiley, 1995.

[40] Reinsel G. Elements of Multivariate Time Series Analysis, Springer, 1997.

[41] Boivin J; Ng S. Understanding and comparing factor-based forecasts. National Bureau of Economic Research; 2005 5 2.

[42] Eurostat, http://ec.europa.eu/eurostat/.

[43] National Institute of Statistics, Romania, http://www.insse.ro/cms/en.

[44] European Central Bank, https://www.ecb.europa.eu/home/html/index.en.html.

[45] National Bank of Romania, http://www.bnro.ro/Home.aspx.

[46] Catell RB. The Scree test for the number of factors, Multivariate Behavioral Research 1/1966. 10.1207/s15327906mbr0102_10 26828106

[47] Bai J; Ng S. Determining the number of primitive shocks in factor models, Journal of Business and Economic Statistics, 2007; 25: 52–60.

[48] Onatski A. Determining the Number of Factors from Empirical Distribution of Eigenvalues, Review of Economic and Statistics, 2010; 92(4): 1004–16.

[49] European Commission, Winter 2017 Economic Forecast. https://ec.europa.eu/info/sites/info/files/ecfin_forecast_winter_1317_ro_en_0.pdf, [retrieved 11 April 2017].

[50] International Monetary, http://www.imf.org/en/News/Articles/2017/03/17/ms031717-romania-staff-concluding-statement-of-the-2017-article-iv-mission [retrieved 11 April 2017].

[51] National Bank of Romania’s semi-annual Report of Financial Stability, http://bnro.ro/files/d/Pubs_ro/RSF/RSF2016dec.pdf [Retrieved 11 April 2017].

[52] Andrei J, Mieila M., Popescu G H., Nica E., Cristina M. The impact and determinants of environmental taxation on economic growth communities in Romania. Energies, 2016; 9(11), 902

[53] Drăgoi MC. The health work force migration: economic and social effects. Farmacia, 2015, 63(4): 593–600.

[54] Popescu G; Boboc D; Stoian M; Zaharia A; Ladaru GR. A cross-sectional study of sustainability assessment. Econ. Comput. Econ. Cybern. Stud. Res. 2017; 51 (1).

[55] Florea NV; Duica A; Duica MC.Using Models and Evaluation Planning to Improve Corporate Training Activity and Trainee Performance. Valahian Journal of Economic Studies, 2016; 7(1),45.

