# Supplementary material for: A multifactor approach to forecasting Romanian gross domestic product (GDP) in the short run
Source: PLoS One. 2017 Jul 24;12(7):e0181379. doi: 10.1371/journal.pone.0181379 (PMC5524352; doi:10.1371/journal.pone.0181379)
Supplement: S2 Table — (DOCX) [file pone.0181379.s002.docx]

**S2 Table**

**VARIABLES INCLUDED IN THE SW_2_**

| **Variable** | **Source** | **Transformation** |
| --- | --- | --- |
| Gross domestic product at market prices | Eurostat | qoq |
| Value added, gross | Eurostat | qoq |
| Final consumption expenditure of general government | Eurostat | qoq |
| Household and NPISH final consumption expenditure | Eurostat | qoq |
| Gross fixed capital formation | Eurostat | qoq |
| Exports of goods and services | Eurostat | qoq |
| Imports of goods and service | Eurostat | qoq |
| Taxes less subsidies on products | Eurostat | qoq |
| Final consumption expenditure of households, durable goods | Eurostat | qoq |
| Final consumption expenditure of households, semi-durable goods, non-durable goods and services | Eurostat | qoq |
| Value added, gross;Agriculture, forestry and fishing | Eurostat | qoq |
| Value added, gross;Industry (except construction) | Eurostat | qoq |
| Value added, gross;Construction | Eurostat | qoq |
| Value added, gross;Wholesale and retail trade, transport, accomodation and food service activities | Eurostat | qoq |
| Value added, gross;Information and communication | Eurostat | qoq |
| Value added, gross;Financial and insurance activities | Eurostat | qoq |
| Value added, gross;Real estate activities | Eurostat | qoq |
| HICP - Overall index excluding energy, food, alcohol and tobacco | Eurostat | qoq |
| HICP - Liquid fuels and fuels and lubricants for personal transport equipment | Eurostat | qoq |
| Current account;Balance | Eurostat | diff q |
| Goods and services;Balance | Eurostat | diff q |
| Financial account; Direct Investment;Net | Eurostat | diff q |
| Financial account; Portfolio Investment;Net positions at the end of the period | Eurostat | qoq |
| Government consolidated gross debt;Percentage of gross domestic product (GDP) | Eurostat | diff q |
| Financial account; Reserve assets;Central Bank | Eurostat | qoq |
| Volume index of production;Mining and quarrying; manufacturing; electricity, gas, steam and air conditioning supply | Eurostat | qoq |
| Index of turnover - Total;Mining and quarrying; manufacturing | Eurostat | qoq |
| Volume index of production;Construction | Eurostat | qoq |
| Building permits - m2 of useful floor area;Buildings | Eurostat | qoq |
| Index of turnover - Total;Wholesale and retail trade;repair of motor vehicles and motorcycles | Eurostat | qoq |
| Index of turnover - Total;Sale of motor vehicles | Eurostat | qoq |
| Index of turnover - Total;Wholesale trade, except of motor vehicles and motorcycles | Eurostat | qoq |
| Index of turnover - Total;Retail trade, except of motor vehicles and motorcycles | Eurostat | qoq |
| Employment (number of persons employed);Construction | Eurostat | qoq |
| Unemployment rate (ILO) | Eurostat | diff q |
| Monetary Aggregate M3 | NBR | qoq |
| Monetary Aggregate M1 | NBR | qoq |
| Monetary Base - Cash | NBR | qoq |
| Monetary Base - Overnight Deposits | NBR | qoq |
| Loan Stock | NBR | qoq |
| Interest on Loans | NBR | diff q |
| Real labour productivity per person | Eurostat | qoq |
| Economy - Gross Salary, RON | INSSE | qoq |
| Industry - Gross Salary, RON | INSSE | qoq |
| Employees, ths persons | INSSE | qoq |
| Employees in Industry, ths persons | INSSE | qoq |
| Construction confidence indicator | Eurostat | diff q |
| Economic sentiment indicator | Eurostat | diff q |
| Industrial confidence indicator | Eurostat | diff q |
| Consumer confidence indicator | Eurostat | diff q |
| Services Confidence Indicator | Eurostat | diff q |
| ROBOR O/N | Eurostat | diff q |
| EUR/RON FX Rate | Eurostat | qoq |
| USD/RON FX Rate | Eurostat | qoq |
| BRENT 1M Forward, EUR/bbl | ECB | qoq |
| BRENT SPOT, USD/bbl | EIA | qoq |
| EA-19 Gross domestic product at market prices | Eurostat | qoq |
| EA-19 Final consumption expenditure | Eurostat | qoq |
| EA-19 Gross fixed capital formation | Eurostat | qoq |
| EA-19 Exports of goods and services | Eurostat | qoq |
| EA-19 Imports of goods and services | Eurostat | qoq |
| EURIBOR 3M | ECB | diff q |
| US Real Gross Domestic Product | St Louis FED | qoq |
| EA-19 Economic sentiment indicator | Eurostat | qoq |
| BVB Stock Market Capitalisation, RON | Eurostat | qoq |

Source: authors` own design
